# Case report of combination therapy with Azacytidine, Enasidenib and Venetoclax in primary refractory AML

**DOI:** 10.1186/s40164-020-00186-y

**Published:** 2021-01-04

**Authors:** Sakshi Jasra, Mohammed Kazemi, Nishi Shah, Jiahao Chen, Karen Fehn, Yanhua Wang, Ioannis Mantzaris, Noah Kornblum, Alejandro Sica, LizaMarie Bachier, Mendel Goldfinger, Kira Gritsman, Ira Braunschweig, Ulrich Steidl, Aditi Shastri, Amit Verma

**Affiliations:** 1 Department of Hematology/Oncology, University of Vermont, 89 Beaumont Avenue, Given E-214, Burlington, VT 05405 USA; 2 Albert Einstein College of Medicine, Department of Hematology/Oncology, Bronx, NY USA

## Abstract

Optimal treatment of acute myeloid leukemia (AML) arising in elderly patients remains a challenge. FDA approval of Ivosidenib and Enasidenib, small molecule inhibitors of isocitrate dehydrogenase enzymes (IDH1 and 2) have opened new avenues of treatment. We present a 60-year-old woman with refractory AML, achieving complete response to the combination therapy of hypomethylating agent, Azacytidine with the IDH2 inhibitor, Enasidenib, and BCL2 inhibitor, Venetoclax. To our knowledge, this is the first case report of a patient with IDH2 mutated refractory AML achieving complete response to combination therapy with azacytidine, enasidenib and venetoclax.

## Letter to the editor

Acute myeloid leukemia (AML) arising in older patients from pre-existing Myelodysplastic Syndrome (MDS) carries a poor prognosis, with limited response to standard cytotoxic therapy [1]. Novel treatments tailored to each patient’s molecular abnormalities are needed to improve outcomes. Mutations in isocitrate dehydrogenase enzymes, IDH1 (R132) and mitochondrial IDH2 (R140, R172) have been identified and lead to the production of an oncometabolite, 2-hydroxyglutarate (2-HG) [2]. Accumulation of 2-HG in hematopoietic stem cells leads to DNA hypermethylation, changes in gene expression and blocked hematopoietic differentiation [2]. Ivosidenib and Enasidenib, small molecule inhibitors of IDH1 and IDH2 enzymes, respectively, are FDA approved for treatment of adults with relapsed/refractory AML [3, 4]. The initial Phase 1/2 open-label, single arm trial of Enasidenib evaluated its efficacy as a single agent in N = 199 patients with relapsed/refractory AML and demonstrated a complete response (CR) of 23% after a median follow-up of 6.6 months [3]. Trials examining these agents in the frontline and relapsed settings, in combination with chemotherapy, are ongoing. We report here a case of a patient with IDH2 mutated refractory AML achieving complete response with the combination of Enasidenib and hypomethylating agent, Azacytidine (AZA). Subsequent relapse was managed with the addition of Venetoclax to the AZA/Enasidenib combination.

Our patient, a 60 year old female with no significant past medical history, presented with worsening fatigue, fever and sore throat. Labs demonstrated a white blood cell (WBC) count of 1.2 × 10^9^/L with an absolute neutrophil count (ANC) of 144 cells/uL, hemoglobin of 12 g/dL, mean corpuscular value (MCV) of 108 fL, platelet count of 187 × 10^9^/L. Initial bone marrow biopsy revealed a hypercellular marrow for age (60% cellularity) with at least 20% CD34 + , CD117 + myeloblasts, concerning for evolving AML. Patient presented to our clinic for a second opinion. Repeat bone marrow biopsy done at this time showed a hypocellular marrow (10–20% cellularity) with at least 40% CD34 + , CD33 + , CD117 + and myeloperoxidase (MPO) + myeloblasts, consistent with AML. Cytogenetic analysis demonstrated trisomy 8. Molecular profiling using a next generation sequencing (NGS) platform revealed a missense mutation in the IDH2 enzyme (c.515G > A; p.R172K) with a variant allele frequency (VAF) of 7%. Patient was also noted to have co-mutations in DNMT3A (VAF 7%).

Patient received induction chemotherapy with the standard “7 + 3” regimen of Cytarabine 100 mg/m2/dose and Idarubicin 12 mg/m2/dose. Despite receiving anti-fungal prophylaxis, post-induction course was complicated by rhinocerebral mucormycosis requiring several sinonasal debridements and a prolonged hospitalization. In an effort to decrease length of neutropenia and avoid further clinical decline, patient received growth factor support with filgastrim. Due to the severity of the infection and her immunocompromised status, patient was given granulocyte infusions. She required a prolonged course of antifungal therapy with posaconazole which patient continues at this time. Upon clinical improvement, repeat bone marrow biopsy was performed on Day 40 post induction. This revealed refractory disease, with 45% CD33 + , CD34 + , CD117 + , and MPO + myeloblasts.

**Fig. 1:**
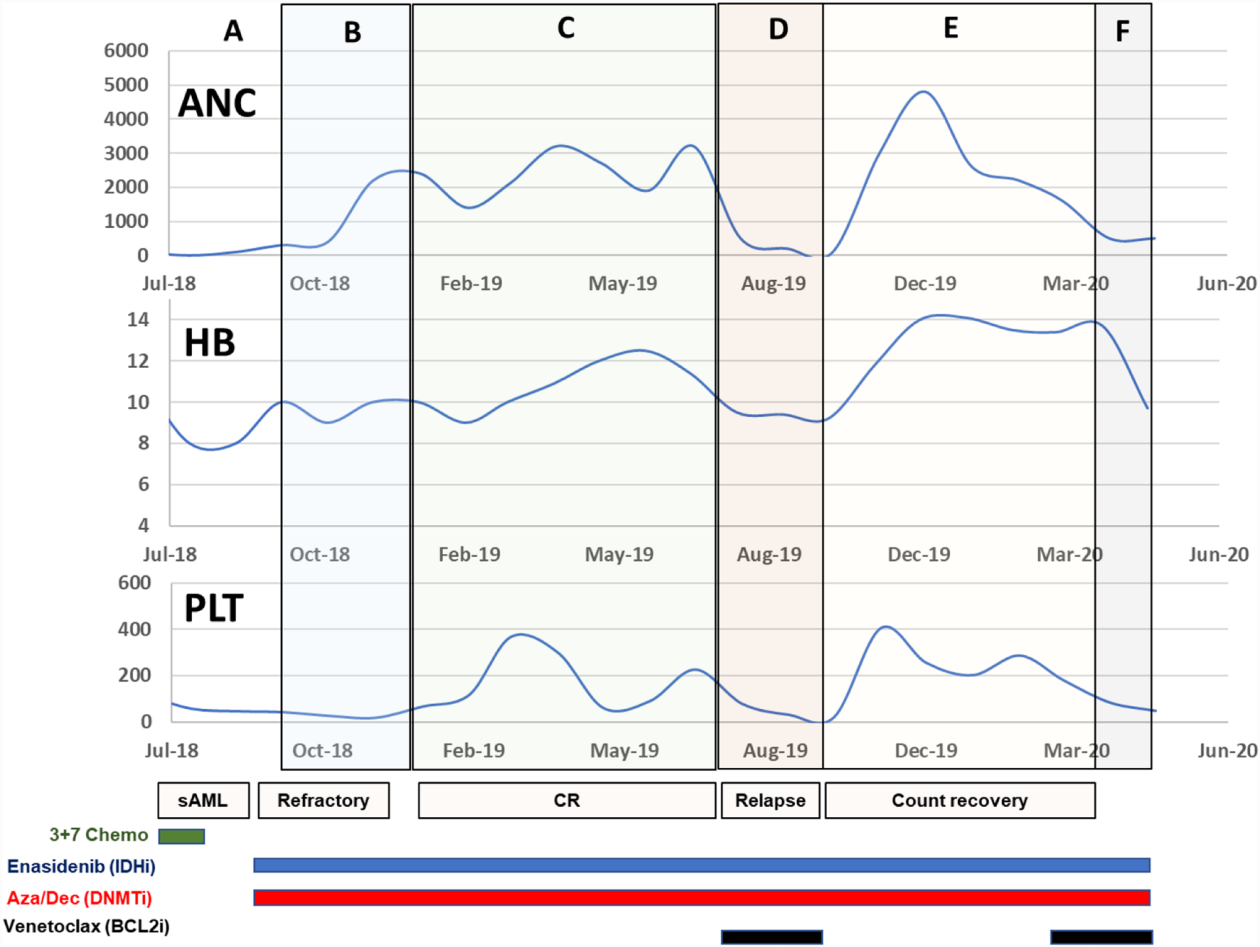
Clinical course of primary refractory AML patient with combination therapy with DNMT inhibitors, IDH inhibitors and Venetoclax: The patient was primary refractory after induction chemotherapy. The patient went into a complete remission (CR) after starting Azacytidine and Enasidenib. Venetoclax was added upon relapse and led to trilineage count recovery. Time periods are as follows: **a** Induction chemotherapy with Idarubicin and Cytarabine; **b** Salvage with Enasidenib and Decitabine; **c** Maintenance with Enasidenib and Azacytidine; **d** Relapse and brief treatment with Venetoclax; **e** Count Recovery; **f** Relapse

We discussed the role of a salvage regimen followed by an allogeneic transplant. However, given her recent invasive fungal infection and poor performance status, patient elected not to pursue a stem cell transplant. She was started on salvage therapy with Enasidenib 100 mg daily in continuous 28 day cycles, and Decitabine 20 mg/m2/dose over five days in 28 day cycles. She did not experience any differentiation syndrome with Enasidenib therapy. However, she developed persistent neutropenia with this regimen and was switched to AZA 75 mg/m2/dose over five days in 28 day cycles. Enasidenib was continued at the initial dose. Figure 1 is a longitudinal representation of the changes in patient’s cytopenias with treatment.

**Fig. 2:**
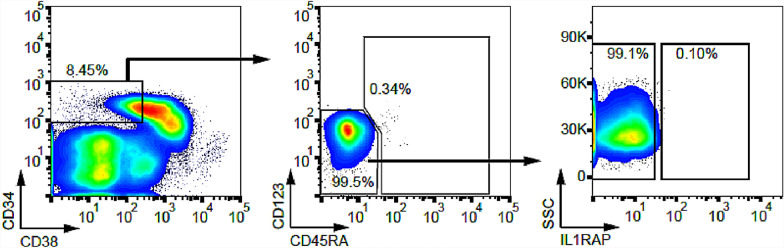
Leukemic stem cells analysis: Bone marrow cells were used to identify Lineage–ve, CD34 + , CD38 −, CD123 + , CD45RA + . IL1RAP + cells with multiparameter flow cytometry. Very few (< 1%) leukemic stem cells were found in remission samples

Bone marrow biopsy performed after seven cycles of Enasidenib and hypomethylating agent therapy showed a hypocellular marrow (10%) with 1.4% CD34 + , CD117 + myeloblasts. Repeat NGS profiling showed a persistent IDH2 R172 clone with a VAF of 10% and a co-mutation in DNMT3A with VAF 12%. Correlative studies with multiparameter flow cytometry revealed less than < 1% phenotypic leukemia stem cells (Lineage-ve, CD34 + , CD38-, CD123 + /CD45RA + /IL1RAP +) demonstrating a deep marrow remission (Fig. 2) [5]. Labs were consistent with complete remission (CR), with a WBC count of 7.3 × 10^9^/L with ANC of 5800 cells/uL, hemoglobin of 10.2, MCV 105 fL and platelets 349 × 10^9^/L.

Patient remained in CR for nine months. She subsequently was noticed to have increasing peripheral blasts. Bone marrow biopsy showed a hypercellular marrow with 10–20% blasts with a new deletion of chromosome 7q22 and a translocation between chromosomes 4 and 13 involving breakpoints -4q22 and 13p12. Sequencing revealed clonal evolution with increasing allele burden of known IDH2 mutation, with VAF now at 38%, and new mutations in BCOR and RUNX1. She was started on Venetoclax at 50 mg daily for 2 weeks. However, patient developed febrile neutropenia and Venetoclax was held. Upon hematologic recovery, clearance of peripheral blasts was noted. Patient was restarted on Venetoclax, AZA and Enasidenib.

This is the first reported case of a patient with relapsed AML responding to combined therapy with a hypomethylating agent (HMA), IDH2 and BCL2 inhibitors. While the two-drug combinations of HMA/Venetoclax and HMA/Enasidenib are currently under investigation, the three-drug regimen of HMA/Venetoclax with Enasidenib has never been evaluated in a clinical trial. The recently published VIALE-A trial has established the combination of AZA/Venetoclax as a viable treatment option in patients with newly diagnosed AML, with a survival benefit noted in the AZA/Venetoclax arm, compared to control (14.7 months vs. 9.6 months, HR 0.66, CI 0.52 to 0.85, p < 0.001) [6]. The combination of AZA/IDH inhibitor is under investigation in the ongoing Phase II AG221-AML-005 trial in patients with IDH2 mutated relapsed/refractory AML. An interim analysis demonstrated an improved CR rate of 53% with AZA/Enasidenib, compared to 12% in the single-agent AZA arm (p = 0.0001) [7]. A Phase III trial of Azacitidine with Ivosidenib for newly diagnosed IDH1 mutated AML patients is also underway (AGILE study, NCT02632708).

The rationale for combining AZA and IDH inhibitors comes from a preclinical model of IDH2-R140Q mutated erythroleukemia cells [8]. RNA-sequencing of cells treated with Enasidenib and Azacitidine showed enrichment of differentiation and cell death gene signatures compared to single drug treated cells. The combination of DNMT and IDH inhibition is hypothesized to lead to a reversal of aberrant epigenetic changes, via two complementary mechanisms. Of note, in our patient, flow-cytometry analysis of bone marrow collected after seven cycles of combination therapy, did not show any remaining leukemic stem cells, as defined by phenotypic markers.

Similarly, other preclinical studies have provided the basis for combining BCL2 and IDH inhibitors. In an RNA interference screen study, IDH mutant AML cells were found to be sensitive to BCL2 inhibition, compared to wild type leukemic cells, due to the accumulation of the oncometabolite, 2-HG [9]. Interestingly, in clinical trials of Venetoclax, the objective response rate appears to be higher in patients with IDH1 or IDH2 mutations, compared to wildtype (33% compared to 10% in IDH-wildtype patients) [10]. There is an upcoming clinical trial investigating the combination of BCL2 and IDH inhibitors (NCT04092179). Taken together, these preclinical studies suggest a rationale for concurrent therapy with HMA/IDH and BCL2 inhibitors. This case is the first report of a patient with relapsed AML achieving clinical benefit with concurrent HMA/IDH/BCL2 inhibition, and merits further investigation in a randomized setting.

Of note, our patient did not experience any differentiation syndrome (DS) after initiation of either Enasidenib or Azacitidine. This phenomenon has been well described in AML patients undergoing therapy with both Enasidenib and Ivosidenib, ranging from within a few days to several months. However, the presence of DS has not been associated with clinical efficacy, contrary to what is observed in Acute Promyelocytic Leukemia (APL) [11]. In fact, studies have demonstrated lower rates of CR in patients with DS, with the reason for this being unclear. Higher peripheral blood blast percentage, fewer lines of previous therapy and an elevated LDH are thought to predict for the risk of DS [11]. In our patient, her initial relapse was soon after induction chemotherapy, and this may have accounted for the lack of DS. However, given the high mortality associated with DS, clinicians are urged to be vigilant, and initiate steroids when suspicion for the same arises.

In conclusion, we present the first report of a patient with refractory AML, ineligible for stem cell transplant, achieving CR with the concurrent three-drug regimen of AZA, Venetoclax and Enasidenib. This novel treatment combination merits further investigation in a clinical trial.
